# The association between dietary inflammatory index with endometriosis: NHANES 2001–2006

**DOI:** 10.1371/journal.pone.0283216

**Published:** 2023-04-26

**Authors:** Pan-Wei Hu, Bi-Rong Yang, Xiao-Le Zhang, Xiao-Tong Yan, Juan-Juan Ma, Cong Qi, Guo-Jing Jiang

**Affiliations:** Department of Gynecology and Obstetrics, Shuguang Hospital Affiliated to Shanghai University of Traditional Chinese Medicine, Shanghai, China; University of Mississippi Medical Center, UNITED STATES

## Abstract

Endometriosis is a common chronic inflammatory and estrogen-dependent disease that mostly affects people of childbearing age. The dietary inflammatory index (DII) is a novel instrument for assessing the overall inflammatory potential of diet. However, no studies have shown the relationship between DII and endometriosis to date. This study aimed to elucidate the relationship between DII and endometriosis. Data were acquired from the National Health and Nutrition Examination Survey (NHANES) 2001–2006. DII was calculated using an inbuilt function in the R package. Relevant patient information was obtained through a questionnaire containing their gynecological history. Based on an endometriosis questionnaire survey, those participants who answered yes were considered cases (with endometriosis), and participants who answered no were considered as controls (without endometriosis) group. Multivariate weighted logistic regression was applied to examine the correlation between DII and endometriosis. Subgroup analysis and smoothing curve between DII and endometriosis were conducted in a further investigation. Compared to the control group, patients were prone to having a higher DII (P = 0.014). Adjusted multivariate regression models showed that DII was positively correlated with the incidence of endometriosis (P < 0.05). Analysis of subgroups revealed no significant heterogeneity. In middle-aged and older women (age ≥ 35 years), the smoothing curve fitting analysis results demonstrated a non-linear relationship between DII and the prevalence of endometriosis. Therefore, using DII as an indicator of dietary-related inflammation may help to provide new insight into the role of diet in the prevention and management of endometriosis.

## Introduction

Endometriosis (EM) is the presence of functional endometrial-similar tissue outside of the uterus, which may lead to the occurrence of many chronic diseases such as pelvic pain, dysmenorrhea, and infertility [1]. As a chronic estrogen-dependent disease, EM has become increasingly prevalent in women, accounting for 6–10%, during reproductive age [2]. In addition to the direct health care costs such as surgery, monitoring tests, and hospitalization, EM also exerts a substantial mental stress regarding infertility [3]. The search for new prevention and management strategies to control EM is crucial.

Food intake is inextricably bound to a person’s life, and a high anti-inflammatory diet reportedly improves the body’s inflammatory status [4]. Several studies have demonstrated that dietary intakes such as red meat, soya, and alcohol are closely involved with the onset of EM [5]. In EM patients, a reasonably balanced diet may be helpful to relieve pain through its interaction with viscerosensory input [6].

The dietary inflammatory index (DII), developed by the University of South Carolina, is widely used to measure the inflammatory potential of a person’s diet [7]. By summing inflammatory index scores across the dietary parameters, an individual’s diet is categorized within a range from anti-inflammatory to pro-inflammatory [8]. Moreover, recent studies suggested that an increased DII not only affects the physical health of the patients, increasing the incidence of endometrial cancer [9], but also has a significant effect on mental health [10]. However, to our knowledge, there is limited information available regarding the correlation between DII and EM. Therefore, with the help of the public data from the National Health and Nutrition Examination Survey (NHANES), this study aimed to examine the connection between DII and EM.

## Materials and methods

### Study population

**Fig 1:**
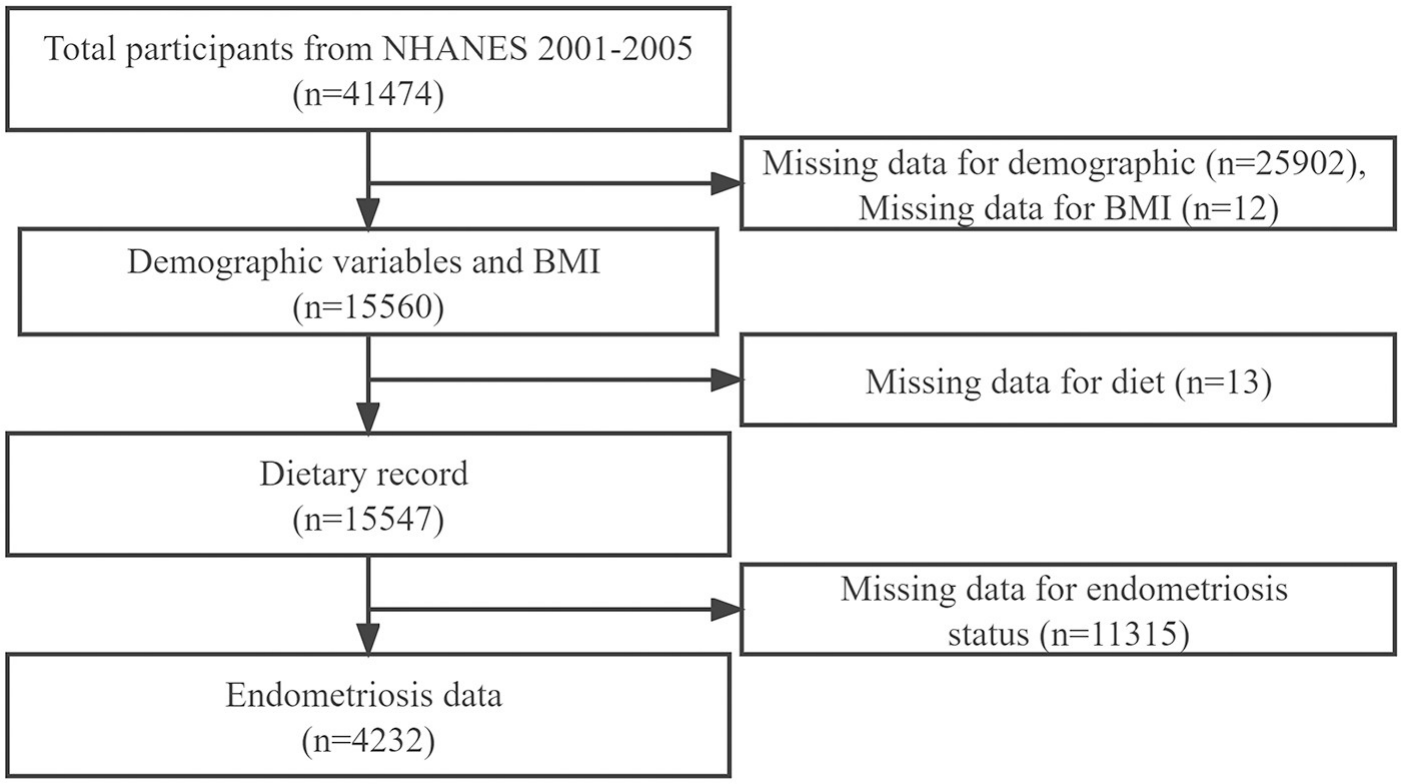
Flowchart of the study population (NHANES 2001–2006). After screening, a total number of 4232 participants were enrolled including 3936 controls (without EM) and 296 cases (with EM). NHANES: National Health and Nutrition Examination Survey.

The NHANES (https://www.cdc.gov/nchs/nhanes) is an ongoing cross-sectional survey that evaluates the diet and health of the noninstitutionalized US civilian population [11]. Using a complex multistage probability sampling design, a representative sample of the U.S. population was selected for the NHANES. Participants were interviewed in their homes, followed by a physical examination and an interview in a mobile examination center. Data for this study were obtained from three consecutive 2-year NHANES cycles (2001–2002, 2003–2004, 2005–2006). Since a complete assessment was not available for all participants, only those who met the predefined criteria were enrolled in our study. The inclusion criteria included complete information on: 1) demographics (including age, race, education, and poverty) and body mass index (BMI) variables 2) raw dietary components 3) EM. A total number of 4232 participants were included in this study (Fig 1). This investigation was granted by the National Health and Nutrition Examination Survey Ethics Review Board, and no external ethic approval was required for this study.

### Study variables

#### Covariates

With reference to recent related studies [12, 13], the following variables were introduced as covariates: age, age at menarche, education (categorized as less high school, high school, and college or above), race (categorized as White, Mexican American, Black and other races), poverty income ratio (PIR), and BMI. Age at menarche was obtained via using the question “How old were you when you had your first menstrual period?”. Demographic data (age, education, race, and PIR) were obtained through a household interview survey. PIR was categorized into three groups: low (< 1.35), medium (1.35 to < 3.0), and high (≥ 3.0) [14]. BMI information was measured in the mobile examination center, and recoded into four categories: underweight (< 18.5 kg/m^2^); normal weight (18.5 to ≤ 24.9 kg/m^2^); overweight (25 to ≤ 29.9 kg/m^2^); obese (> 30 kg/m^2^).

#### DII

As a literature-based scoring algorithm, the DII is now widely used as a special parameter for evaluating overall dietary inflammation. Based on the effect of 45 different food parameters on inflammatory biomarkers, an individual diet’s overall inflammatory potential score was ranked from the most anti-inflammatory to the most pro-inflammatory [15]. Due to the inherent restrictions of the NHANES data, dietary intake was only available for 28 out of the 45 food parameters [16]. However, because vitamin D intake data collection began in 2007, only 27 dietary parameters were enrolled in this study including: carbohydrates; protein; total fat; alcohol; fiber; cholesterol; saturated fat; monounsaturated and polyunsaturated fatty acids; n-3 and n-6 polyunsaturated fatty acids; niacin; vitamins A, B1, B2, B6, B12, C, D, E; iron; magnesium; zinc; selenium; folic acid; beta carotene; caffeine; energy. Through conducting a 24-h dietary recall interview at the mobile examination center, 27 components of each participant consumed in a 24-h period were acquired. DII contained in the diet was acquired using an inbuilt function in the ‘nhanesR’ package. By subtracting the standard mean of each food parameter and dividing it by the standard deviation, the individual’s Z-score value was measured and converted to centered proportions. They were multiplied by the responding inflammatory effect index and summed up, yielding a total DII score for each participant [17]. For further analyses, the DII was equally divided into four groups, ranging from the lowest quartile (maximally anti-inflammatory) to the highest quartile (maximally pro-inflammatory).

#### EM

EM was diagnosed based on a questionnaire-based survey asking: “Has a doctor or other health professional ever told you that you had endometriosis (age at interview 20–54 years)?” Those participants who answered yes were considered cases. Participants who answered no were considered controls.

### Statistical analysis

All data were analyzed via R statistical (version 4.1.1) and EmpowerStats software (http://www.empowerstats.net). For an impartial evaluation, weighted data were analyzed based on the available guidelines of NHANES. For continuous variables, statistics were calculated using weighted logistic regression to generate P values. For categorical variables, P values were determined by a weighted chi-square test. Three multiple weighted logistic regression models were developed to explore the relationship between DII and EM: the crude model was not adjusted for any covariate; model 1 was adjusted for age and race; model 2 was adjusted for all covariates. A smoothing curve was performed to visually estimate the association between DII and EM. P < 0.05 was considered statistically significant for all analyses.

## Results

### Participant characteristics

A total of 4232 participants were enrolled, comprising 3936 controls and 296 cases. Table 1 displays the baseline characteristics of participants in each group. Compared to the controls, the case group was more likely to have a higher distribution of DII (pro-inflammatory diet), age, education, and PIR. In our study, the whites were more prone to suffer EM than other races.

**Table 1:** Weighted baseline characteristics of participants.

	Controls (n = 3936)	Cases (n = 296)	P-value
N	3936	296	
DII	1.82±1.66	2.01±1.68	0.014
Age, years	35.28±10.11	40.42±8.51	< 0.001
Age at menarche, years	12.58±1.70	12.40±1.82	0.211
BMI, n (%)			0.854
Underweight	93 (2.41%)	6 (2.07%)	
Normal weight	1234 (32.04%)	101 (34.83%)	
Obese	1432 (37.18%)	106 (36.55%)	
Overweight	1093 (28.37%)	77 (26.55%)	
Race, n (%)			< 0.001
White	1825 (46.37%)	202 (68.24%)	
Black	846 (21.49%)	56 (18.92%)	
Mexican American	905 (22.99%)	24 (8.11%)	
Other races	360 (9.15%)	14 (4.73%)	
Education, n (%)			0.002
Less high school	923 (23.46%)	38 (12.84%)	
High school	852 (21.66%)	79 (26.69%)	
College or above	2159 (54.88%)	179 (60.47%)	
PIR, n (%)			0.03
Low	1153 (30.72%)	63 (21.88%)	
Medium	1075 (28.64%)	64 (22.22%)	
High	1525 (40.63%)	161 (55.90%)	

Values are presented as mean ± SD for continuous variables, and P-value was calculated by the weighted linear regression. Values are presented as percent (%) for categorical variables, and P-value was calculated by weighted chi-square test.

DII: dietary inflammatory index; BMI: body mass index; PIR: poverty income ratio.

### Association between DII and EM

Weighted generalized logistic regression analysis was performed to probe the relationship between DII and EM, and a positive correlation was identified (Table 2). When compared to the lowest quartile of DII, the fourth quartile had a higher prevalence of EM in the crude model [Odds ratio (OR) = 1.43, 95% confidence interval (CI) = 1.00–2.05, P = 0.059], model 1 (OR = 1.54, 95% CI = 1.08–2.20, P = 0.022), and model 2 (OR = 1.61, 95% CI = 1.09–2.37, P = 0.018). With the escalation of DII in all models, the OR for EM increases as well (P for trend < 0.05).

**Table 2:** The odds ratio for the relationship between DII and EM.

	Crude, OR (95% CI, P)	Model 1, OR (95% CI, P)	Model 2, OR (95% CI, P)
DII	1.10 (1.02, 1.20) 0.023	1.12 (1.04, 1.22) 0.007	1.12 (1.03, 1.22) 0.013
Quartile of DII			
Q1	1 (Reference)	1 (Reference)	1 (Reference)
Q2	0.89 (0.55, 1.43) 0.631	0.91 (0.56, 1.49) 0.712	0.92 (0.55, 1.55) 0.747
Q3	1.41 (0.96, 2.08) 0.091	1.53 (1.03, 2.27) 0.043	1.41 (0.90, 2.21) 0.124
Q4	1.43 (1.00, 2.05) 0.059	1.54 (1.08, 2.20) 0.022	1.61 (1.09, 2.37) 0.018
P for trend	0.014	0.003	0.006

Crude: no covariates were adjusted; Model 1: only age and race were adjusted; Model 2: age, age at menarche, BMI, race, education, and PIR were adjusted.

DII: dietary inflammatory index; EM: endometriosis; BMI: body mass index; PIR: poverty income ratio; OR: odds ratio; CI: confidence interval.

### Subgroup analysis

To resolve the heterogeneity between groups, subgroup analysis was conducted for different covariates (age, race, education, and PIR). Participants older than 35 years were more prone to suffer EM in all models (P = 0.005, P = 0.003, P = 0.019), while those under 35 years, did not show a statistically significant association (P = 0.595, P = 0.609, P = 0.652). For participants with EM in model 2, positive and significant associations were observed in the white (OR = 1.17, 95% CI = 1.05–1.31, P = 0.007), college or above education (OR = 1.22, 95% CI = 1.05–1.41, P = 0.013) and high PIR (OR = 1.19, 95% CI = 1.01–1.39, P = 0.041) groups. In contrast, among Black (OR = 1.02, 95% CI = 0.75–1.37, P = 0.914), Mexican American (OR = 1.14, 95% CI = 0.94–1.38, P = 0.189), and other races (OR = 1.16, 95% CI = 0.65–2.06, P = 0.614), the relationship between DII and EM was not statistically significant in model 2. The same conclusion was found in participants with low education (OR = 1.19, 95% CI = 0.98–1.46, P = 0.095) or poor economic conditions (OR = 1.01, 95% CI = 0.86–1.19, P = 0.918). Meanwhile, no interaction with DII was detected in model 2 (P for interaction > 0.05), indicating that the correlation between DII and EM did not vary by age, race, education, or PIR (Table 3).

**Table 3:** The results of subgroup analyses were stratified by age, race, education, and poverty.

	Crude, OR	Model 1, OR	Model 2, OR
	(95% CI, P)	(95% CI, P)	(95% CI, P)
Age			
< 35 years	1.03 (0.92, 1.17) 0.595	1.03 (0.92, 1.16) 0.609	1.03 (0.90, 1.19) 0.652
≥ 35 years	1.20 (1.06, 1.35) 0.005	1.21 (1.08, 1.37) 0.003	1.16 (1.03, 1.31) 0.019
P for interaction	0.459	0.400	0.346
Race			
White	1.04 (0.93, 1.17) 0.466	1.07 (0.96, 1.19) 0.209	1.17 (1.05, 1.31) 0.007
Black	1.40 (1.13, 1.73) 0.004	1.41 (1.13, 1.75) 0.004	1.02 (0.75, 1.37) 0.914
Mexican American	0.98 (0.82, 1.17) 0.786	0.98 (0.81, 1.18) 0.827	1.14 (0.94, 1.38) 0.189
Other races	1.20 (0.87, 1.67) 0.274	1.20 (0.87, 1.65) 0.273	1.16 (0.65, 2.06) 0.614
P for interaction	0.061	0.047	0.051
Education			
Less high school	1.04 (0.91, 1.19) 0.535	1.04 (0.91, 1.18) 0.604	1.19 (0.98, 1.46) 0.095
High school	1.12 (0.95, 1.32) 0.172	1.16 (0.98, 1.37) 0.092	0.96 (0.83, 1.12) 0.620
College or above	1.15 (0.98, 1.36) 0.100	1.18 (1.00, 1.40) 0.053	1.22 (1.05, 1.41) 0.013
P for interaction	0.128	0.379	0.407
PIR			
Low	1.19 (1.02, 1.38) 0.027	1.22 (1.05, 1.43) 0.015	1.01 (0.86, 1.19) 0.918
Medium	1.11 (0.95, 1.30) 0.193	1.12 (0.96, 1.32) 0.169	1.17 (0.97, 1.41) 0.105
High	1.00 (0.89, 1.13) 0.947	1.02 (0.90, 1.16) 0.750	1.19 (1.01, 1.39) 0.041
P for interaction	0.222	0.608	0.567

Crude: no covariates were adjusted; Model 1: only age and race were adjusted; Model 2: age, age at menarche, BMI, race, education, and PIR were adjusted.

PIR: poverty income ratio; OR: odds ratio; CI: confidence interval.

*Stratified variables themselves were also not adjusted in the subgroup analysis.

### Smoothing curve fitting analysis

**Fig 2:**
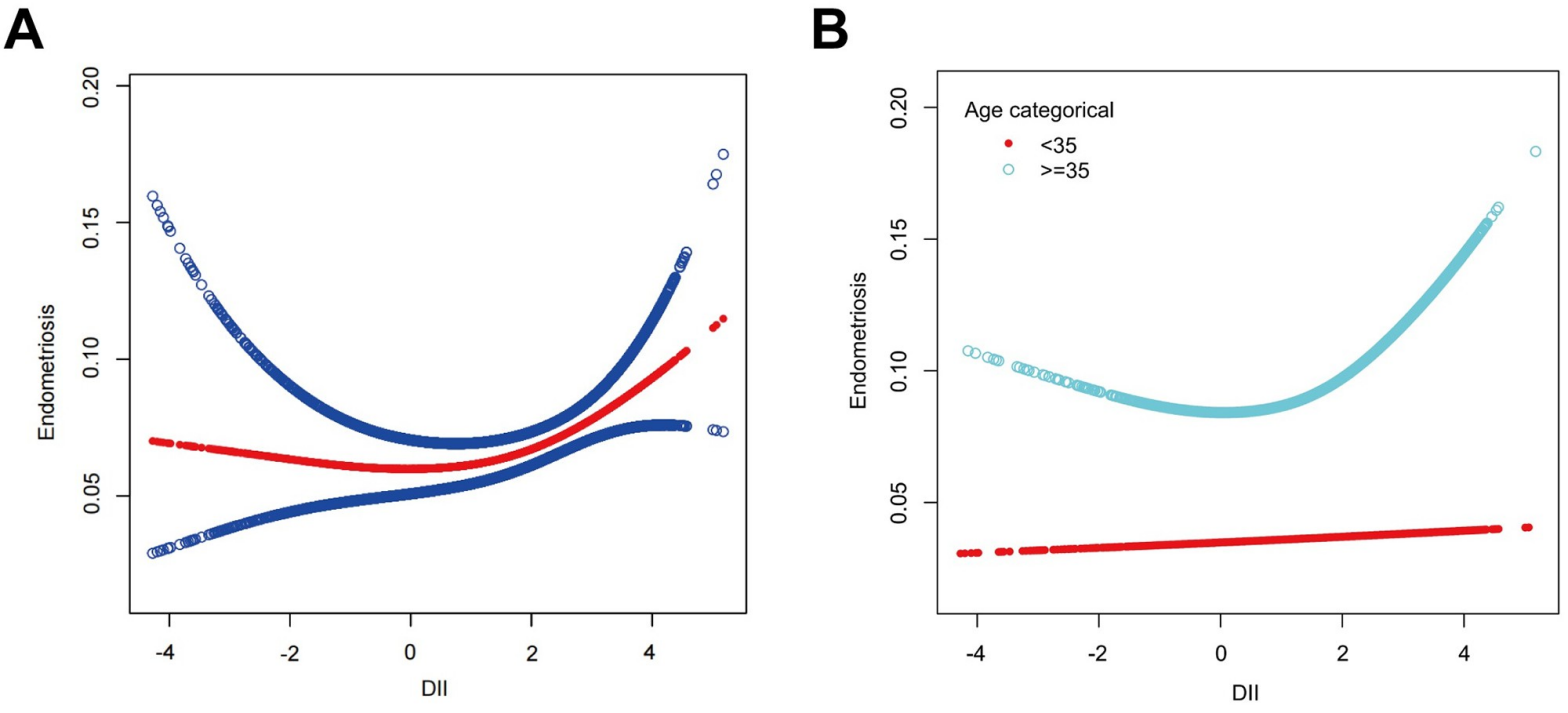
Relationship between DII and EM. (A) The red line represents the correlation between DII and EM, and the blue line stands for the 95% CI (adjusted for age, age at menarche, BMI, race, education, and PIR). (B) In age-stratified analysis, the red line represents age < 35 years while the blue line represents age ≥ 35 years (adjusted for age at menarche, BMI, race, education, and PIR). DII: dietary inflammatory index; EM: endometriosis; BMI: body mass index; PIR: poverty income ratio; CI: confidence interval.

After adjusting for all covariates (model 2), smoothing splines were used to visually demonstrate the relationship between DII and EM. From the overall smoothing curve, a nonlinear relationship between DII and EM (log-likelihood ratio = 0.018) was revealed. When the DII (> 1.76) increased, the odds of EM prevalence increased (OR = 1.33, 95% CI = 1.11–1.60, P = 0.002). Given the critical value of the age category, the results were then stratified by age (< 35, ≥ 35) for further analysis. For participants aged < 35 years, the association trend was not statistically significant (OR = 1.04, 95% CI = 0.90–1.21, P = 0.596). However, a significant nonlinear relationship (log-likelihood ratio = 0.020) was identified in those aged ≥ 35 years (OR = 1.11, 95% CI = 1.01–1.22, P = 0.036); the probability of EM prevalence increased (OR = 1.35, 95% CI = 1.12–1.64, P = 0.002) as per one unit change in the DII (> 1.47) (Fig 2 and Table 4).

**Table 4:** Threshold analysis between DII and EM.

	Total	Age (< 35 years)	Age (≥ 35 years)
	OR (95% CI, P)	OR (95% CI, P)	OR (95% CI, P)
Standard logistic model	1.09 (1.00, 1.18) 0.038	1.04 (0.90, 1.21) 0.596	1.11 (1.01, 1.22) 0.036
Two-piecewise logistic model			
Inflection point (K)	1.76	3.25	1.47
DII < K	0.94 (0.82, 1.08) 0.417	1.01 (0.85, 1.19) 0.948	0.91 (0.76, 1.09) 0.324
DII > K	1.33 (1.11, 1.60) 0.002	1.58 (0.57, 4.38) 0.375	1.35 (1.12, 1.64) 0.002
Log-likelihood ratio	0.018	0.424	0.020

Age, age at menarche, BMI, race, education, and PIR were adjusted.

DII: dietary inflammatory index; EM: endometriosis; BMI: body mass index; PIR: poverty income ratio; OR: odds ratio; CI: confidence interval.

## Discussion

To our knowledge, this is the first cross-sectional study to identify a positive association between DII and EM. Importantly, this relationship remained significant after adjusting for other exposure covariates in the multivariate regression analysis.

As a common gynecological disorder with high prevalence, EM seriously affects people of childbearing age [18]. While inflammation is a key physiological and pathological feature of EM, the altered inflammatory microenvironment in endometriotic tissue may lead to the persistence of chronic pain [19, 20]. DII, as an objective and standard protocol, is used to effectively monitor the inflammatory potential of an individual’s diet [21]. In a previous study, after 2 weeks of an anti-inflammatory dietary intervention, such as eating vegetables, fiber, and phytochemicals, a significant decrease in pro-inflammatory NF-κB levels was detected in the obese women, meanwhile, the anti-inflammatory cytokine IL-10 increased considerably [22]. A cohort study in Japan also indicated that eating a variety of anti-inflammatory foods, such as vegetables, was recommended; this may ultimately alter perinatal mortality and morbidity among EM patients [23]. Given the importance of inflammation in mediating inflammatory responses in EM, inflammatory dysfunctions have emerged as an attractive target for therapies, providing opportunities for developing alternative disease prevention and treatment [24].

In our study, DII had a significant positive correlation with EM, and a non-linear relationship was obtained using a smoothing spine. To explore this, subgroup analyses were further conducted. In those aged under 35 years old, the effect of DII was less pronounced, while in the older age group, the OR of EM showed a positive linear association with DII (at DII > 1.47). As an estrogen-dependent chronic inflammatory disease, the highest risk of EM is between 25–35 years old [25]. The density of serum hormonal levels, especially estrogen concentrations, typically declines at 35 years old [26], thus, it is speculated that estrogen may act as a predisposing factor associated with the prevalence of EM in young women. With increasing age, the pathogenic role of estrogen decreased, and chronic inflammatory processes gradually became a major precipitating factor. Therefore, DII might also exert a potentially large impact on middle-aged and older women through its interactions with inflammation. Moreover, one additional cross-sectional study of NHANES also demonstrated a non-linear relationship between DII and sex hormones including estrogen in postmenopausal women [27], which might also explain the role of DII in older women. Interestingly, this may be due to racial bias meaning that more white people typically get diagnosed [28] and the DII may play a different role in the prevalence of EM among ethnic groups due to ethnic-specific genetic factors. Participants with EM also tend to have a high income and educational status [29], which was consistent with our results. However, no study provides direct evidence to elucidate the demographic role in the association between DII and EM, and more research is needed in future studies. Collectively, this study highlights the important role of DII in EM; therefore, may hold promise for dietary regimen interventions to help manage the disease in a non-pharmaceutical way.

However, there are some limitations of the current study that should be addressed. First, this study is cross-sectional, and thus a causal relationship could not be demonstrated. Second, given the small sample size we were unable to consider all potential confounding factors such as family history of EM, history of pelvic surgery, pelvic pain, and so on, which may cause bias in the results. Third, as the age of some participants younger than 20 were not enrolled in the diagnosis questionnaires of EM, the results from the present study might not apply to younger patients. Fourth, most of the data used were collected by interviews or self-report questionnaires, as such the control group may inevitably have undiagnosed EM disease, which may lead to recall and report bias. Finally, there is a possibility of sampling error due to the selection of the study population from NHANES.

## Conclusions

In middle-aged and older women (aged ≥ 35 years), exists a non-linear relationship between DII and EM. This may provide new insight into the role of diet in the prevention and treatment of EM. However, further studies are needed to identify the causal relationship and intrinsic mechanism between DII and EM.
